# Concomitant Transverse Myelitis and Acute Axonal Sensory-Motor Neuropathy in an Elderly Patient

**DOI:** 10.1155/2017/7289474

**Published:** 2017-07-13

**Authors:** L. M. Oliveira, R. G. Cury, L. H. Castro, R. Nitrini

**Affiliations:** Department of Neurology, University of Sao Paulo, Sao Paulo, SP, Brazil

## Abstract

Diagnosing concomitant transverse myelitis (TM) and Guillain-Barré syndrome (GBS) can be challenging. We report a case of an elderly patient presenting with acute sensory and motor disturbances in the four limbs, associated with urinary retention, ophthalmoparesis, facial weakness, and dysarthria. Electrodiagnostic studies were consistent with acute motor sensory axonal neuropathy (AMSAN), and imaging showed a longitudinally extensive tumefactive contrast-enhancing hyperintense spinal cord lesion extending from T6 to the cone. Concomitant AMSAN and TM have not been previously reported in the elderly. Comorbid TM and other GBS variants have been previously reported. Intravenous methylprednisolone, plasma exchange, cyclophosphamide, or combination therapies are usually used, although there are no randomized controlled studies regarding treatment choices.

## 1. Introduction

Overlapping acute polyradiculoneuritis and transverse myelitis (TM) represents a diagnostic challenge. Few cases of this simultaneous association have been reported. Association should be suspected when, in addition to Guillain-Barré syndrome features, marked persistent weakness asymmetry, urinary retention at onset, fever, and a sensory level are present [1]. We report a case of concomitant acute motor sensory axonal neuropathy (AMSAN) and transverse myelitis (TM) in a 64-year-old patient. To our knowledge, this is the first case of an elderly presenting with both conditions.

## 2. Case Report

**Figure 1:**
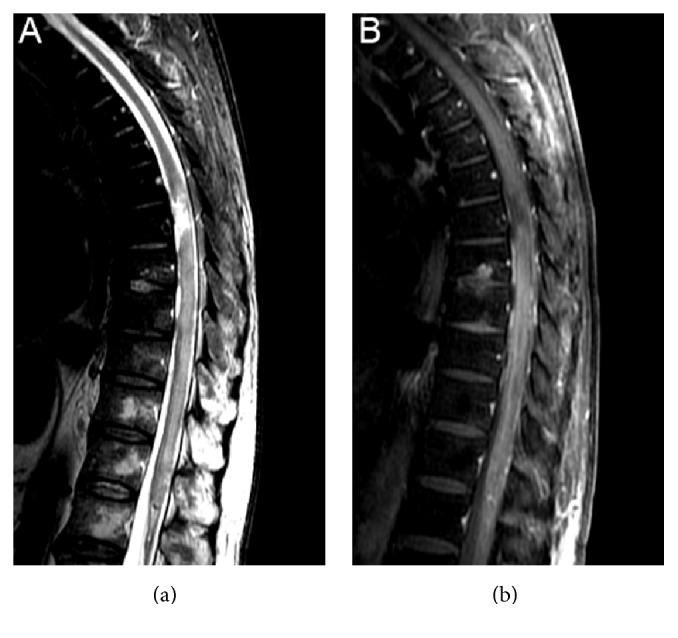
Spinal cord magnetic resonance imaging. Sagittal T2-weighted MRI shows a high signal intensity lesion on T6-T7 levels and a longitudinally extensive tumefactive hyperintense lesion from T6 to the medullary cone (a) with enhancing on postcontrast series (b).

A 64-year-old man presented to the emergency department with a one-day history of lower extremities distal numbness, rapidly progressing to four-limb weakness, back pain, dysarthria, facial weakness, and diplopia. He had received influenza vaccination 18 days before. Five days after vaccination, the patient developed productive cough and diarrhea lasting 3 days. Initial neurological evaluation disclosed marked leg (1/5 grade) and mild arm weakness (4/5 grade), hypoactive deep tendon reflexes, normal plantar reflexes, decreased pinprick, and vibration sensation in all limbs, without a well-defined sensory level, as well as bilateral facial weakness, dysphonia, dysphagia, and restricted eye movements in all directions. The patient quickly developed urinary retention, labile blood pressure, and respiratory distress, ultimately requiring ventilatory support. Brain computed tomography results were normal. Cerebrospinal fluid (CSF) analysis revealed 1 leukocyte/mm^3^, high protein content (763 g/dl), normal glucose level, and negative bacteriological testing. Guillain-Barré syndrome was suspected, and the patient was treated with intravenous immunoglobulin (0.4 mg/kg/d) for 5 days. Electrodiagnostic studies showed decreased motor and sensory potentials, mild slowing of nerve conduction, and denervation signs in all limbs, consistent with an acute axonal motor sensory polyneuropathy, with a markedly predominant motor impairment. Upper limb strength, facial weakness, and eye movements gradually improved. On hospital day 55, the patient no longer required ventilatory support but still experienced urinary retention and bilateral leg anesthesia. A defined T8 sensory level became apparent. A spinal cord MRI showed a longitudinally tumefactive spinal lesion extending from T6 to the cone (Figure 1). Repeat CSF analysis showed increased cell count (31 leukocytes/mm^3^), elevated protein levels (1,722 g/dl), and high gamma globulin levels (29.9%, range 7–14%). CSF polymerase chain reactions (PCR) for* Mycobacterium tuberculosis*, Herpes simplex (HSV) I/II, cytomegalovirus, Epstein-Barr (EBV), and varicella-zoster viruses were negative. Serological testing did not indicate recent or active Borrelia, Bartonella, EBV, HSV I/II, HIV, HTLV, syphilis, hepatitis, or* Mycoplasma pneumoniae* infection. Serum anti-aquaporin-4 and rheumatological tests were negative.

The patient was treated with a 5-day methylprednisolone course (1 g/day), 1 g cyclophosphamide, and six plasma exchange sessions, without lower limb strength or sensation improvement. Additionally, the patient received a 14-day doxycycline course, due to positive* Mycoplasma *serum IgG titers and the history of productive cough. The patient was discharged to long-term rehabilitation on hospital day 170, still unable to walk, on intermittent bladder catheterization.

## 3. Results and Discussion

Association of Guillain-Barré syndrome and TM is uncommon. When described, this association is more commonly related to acute motor axonal neuropathy (AMAN) [2]. We present a case of an elderly patient with concomitant AMSAN and TM. We identified only two previous reports of AMSAN and TM in younger patients: a 28-year-old woman [3] and a pediatric case [4], both with incomplete recovery.

Differentiating classical GBS from the association of polyradiculoneuritis and myelitis can be difficult on initial evaluation. Our patient presented bladder dysfunction at onset that persisted throughout the disease course. Other features that should cast doubt about the diagnosis of GBS include severe respiratory dysfunction or severe sensory signs with limited limb weakness at onset, marked persistent weakness asymmetry, fever at onset, increased CSF cell count, and a sharp spinal cord sensory level [1]. Our patient developed a severe condition within 24 hours after symptom onset. Rapid progression is frequently reported in overlapping TM and GBS cases [5]. The diagnosis of TM was delayed due to infectious complications and difficult mechanical ventilation weaning that limited neurological evaluation.

In a review study evaluating cases of overlapping GBS and acquired demyelinating syndrome [5], 16/21 (76%) of the simultaneous GBS and transverse myelitis cases were preceded by infections or vaccinations. Thirteen patients received immune therapy, mostly prednisone and intravenous immunoglobulin. Outcome was favorable in 11/20 (55%) patients. In our case, influenza vaccination, respiratory symptoms, and a diarrheal disease preceded the neurologic disease and were probably related to the pathophysiology of the overlap syndrome. Although a causal role of* Mycoplasma* infection in GBS and myelitis cannot be established in our case, we opted for a doxycycline course, considering the productive cough history and positive IgG* Mycoplasma* serology. The role of antibiotic therapy in isolated MT or GBS cases associated with* Mycoplasma* is uncertain, since the underlying pathogenic mechanism is incompletely understood, and there are no controlled studies assessing antibiotic use in this setting [3].

## 4. Conclusion

Overlapping cases of acute polyradiculoneuritis and TM constitutes a diagnostic challenge. To our knowledge, there are currently no other records describing the TM/AMSAN association in the elderly. Further reports may help to understand the pathophysiological mechanisms underlying this overlap syndrome and may also indicate better treatment approaches.
